# Comparative Study on Cardiac Findings in Patients with Transthyretin Amyloidosis Before and After Treatment with a Transthyretin Silencer

**DOI:** 10.3390/jcdd12090342

**Published:** 2025-09-05

**Authors:** Priya Arivalagan, Diego Hernan Delgado

**Affiliations:** 1 Division of Cardiology, Peter Munk Cardiac Centre, Toronto General Hospital, University Health Network, University of Toronto, 585 University Ave, PMB11-136, Toronto, ON M5G 2N2, Canada; 2 Toronto General Hospital Research Institute, University Health Network, Toronto, ON M5G 2C4, Canada; 3 Institute of Medical Science, University of Toronto, Toronto, ON M5S 1A8, Canada

**Keywords:** transthyretin amyloid cardiomyopathy, cardiac amyloidosis, inotersen, patisiran, PYP scan, PYP grade, H/CL ratio

## Abstract

Transthyretin amyloidosis (ATTR) is a rare disease caused by misfolded proteins, amyloids, that are deposited in various organs and tissues, typically the heart and/or nerves, causing the development of cardiomyopathy (CM) and polyneuropathy (PN). Although this may be an incurable disease, there are various treatments that are currently available for patients with ATTR, including transthyretin (TTR) silencers such as inotersen and patisiran. The silencers help slow down the progression of disease and improve the quality of life of patients with ATTR by alleviating the cardiac and neurological symptoms that patients present. The purpose of this study was to compare the cardiac findings observed in the ^99^Tc-PYP scintigraphy (PYP scan) parameters of patients with a mixed phenotype before and after treatment with inotersen or patisiran. This study included ten patients from the amyloidosis clinic at the University Health Network. All of the patients (average age: 63.80 ± 11.70; 60.0% males, 40.0% females) received inotersen or patisiran as their treatment. These patients underwent a PYP scan before and after treatment to observe any improvements in terms of their CM post-treatment. Nine (90.0%) patients showed an improvement with their CM, as they showed a decrease in their heart-to-contralateral lung (H/CL) ratio and/or pyrophosphate (PYP) grade based on their results from the PYP scan post-treatment with a TTR silencer. Only one patient (10.0%) had worsening results, as their H/CL ratio and PYP grade increased post-treatment in comparison to the PYP scan results pre-treatment. Patients with ATTR who have a mixed phenotype should undergo a PYP scan before and after treatment with a TTR silencer. By undergoing these scans, the effectiveness of this treatment could be determined by observing any improvements in the signs of CM. A decrease in the H/CL ratio and/or the PYP grade would indicate that the TTR silencer has been effective in alleviating the signs and symptoms of CM, and that the patients should continue with their treatment plan.

## 1. Introduction

Transthyretin amyloidosis (ATTR) is a rare, yet progressive, disease that is caused by misfolded transthyretin (TTR) proteins [1]. TTR proteins are produced in the liver and deposited in other organs and tissues, especially the heart and nerves [2]. The accumulation of TTR proteins results in the presentation of the signs and symptoms of cardiomyopathy (CM) and/or polyneuropathy (PN) [3,4]. Patients who present with only signs and symptoms of CM have transthyretin amyloid cardiomyopathy (ATTR-CM), patients who present with only signs and symptoms of PN have transthyretin amyloid polyneuropathy (ATTR-PN), and patients who present with signs and symptoms of both CM and PN are considered to have ATTR with a mixed phenotype. ATTR-CM tends to be the most prominent diagnosis provided to patients [3]. However, as this is a progressive disease, patients would eventually develop a mixed phenotype [5]. In addition to direct damage caused by transthyretin amyloid fibrils, the deposition of non-fibrillar transthyretin oligomers may participate in the process of tissue damage [6].

There are several diagnostic methods for ATTR-CM. One of these is ^99^technetium pyrophosphate scintigraphy (PYP scan) [7]. A PYP scan is used to specifically diagnose ATTR-CM. A PYP scan provides the following information: a heart-to-contralateral lung ratio (H/CL ratio) and a PYP grade. An H/CL ratio calculates the mean uptake in a region of interest (ROI) over the heart divided by the measurement of a similar-sized ROI in the contralateral lung. An H/CL ratio less than 1.5 or a ratio less than 50% suggests that the patient is negative for ATTR-CM, whereas a ratio greater than 1.5 or a ratio of more than 50% suggests that the patient is positive for ATTR-CM [6,7]. The PYP grade is an indicator of how much uptake of the technetium pyrophosphate radiotracer is found in the myocardium [8]. PYP grades range from zero to three; grade zero suggests that there is no amyloid uptake in the myocardium, grade one suggests that the amyloid uptake in the myocardium is less than the rib uptake, and grades two and three indicate that the amyloid uptake in the myocardium is equal to or greater than the rib uptake [9]. Patients are diagnosed with ATTR-CM if they have an H/CL ratio greater than 1.5 and a PYP grade of two or three [7,9].

Although this disease is progressive and incurable, there are treatments available to improve quality of life and slow down the disease progression. Novel therapeutics that have been effective at alleviating symptoms are TTR silencers [10]. Two types of silencers are inotersen and patisiran. TTR silencers prevent the transcription of the TTR messenger ribonucleic acid (mRNA) by degrading it, thus reducing the formation of TTR proteins, which then reduces the amount of TTR amyloid fibrils depositing in the heart and/or nerves [10,11].

Inotersen and patisiran were approved by Health Canada in 2018 and 2019, respectively, to treat patients with ATTR who present with only the PN phenotype or a mixed phenotype [12,13]. These therapeutics are known to be very effective at alleviating the signs and symptoms of PN and relatively effective at alleviating the signs and symptoms of CM [12,13]. Currently, there are no studies that have compared cardiac effects before and after treatment with a TTR silencer in patients with a mixed phenotype. Therefore, this study aimed to compare the cardiac findings in the PYP scan results among patients with ATTR with a mixed phenotype before and after treatment with inotersen or patisiran.

## 2. Methods

### 2.1. Study Design

This was a single-centre retrospective study that included 10 patients from the amyloidosis clinic at the Peter Munk Cardiac Centre (PMCC) in Toronto, ON, Canada. Patients (>18 years of age) with ATTR who had a mixed phenotype and were being treated with a TTR silencer were included in this study. An ECHO was taken by a cardiologist, demonstrating thickness in the left ventricle and a decrease in the left ventricular ejection fraction. All the patients underwent a blood test, demonstrating an increase in the levels of cardiac biomarkers (i.e., troponin and BNP). A PYP scan was taken by a nuclear cardiologist for each patient before and after treatment with a TTR silencer. Furthermore, a genetic test confirming the presence of a TTR mutation determined ATTR and ruled out AL amyloidosis.

### 2.2. Technetium Pyrophosphate Scintigraphy

Technetium pyrophosphate scintigraphy (PYP scan) is a diagnostic test specific for ATTR-CM [7]. To initiate the test, a 740 MBq technetium pyrophosphate radiotracer is injected to bind to TTR amyloids [9]. Then, a scan is conducted 3 h post-injection of the radiotracer [14]. The PYP scan obtains 3D images of the heart by using dual-head, single-photon emission computed tomography [15]. A nuclear cardiology specialist quantifies the amount of amyloid uptake in the myocardium by assessing the following: (1) the cardiac retention by semi-quantitative visual scoring (grade 0 = no cardiac uptake, grade 1 = mild uptake less than bone, grade 2 = moderate uptake equal to bone, or grade 3 = high uptake greater than bone) and (2) the heart-to-contralateral lung (H/CL) ratio uptake by performing a quantitative analysis [7,8].

### 2.3. Echocardiography

Echocardiography is a diagnostic test that can be used to determine if patients show features of ATTR-CM by evaluating the structural components of the heart [16]. An echocardiogram assesses the heart function through several parameters. A few include the ejection fraction percentage (EF%), which measures the percentage of the heart pumped out from the left ventricle, and the interventricular septal end diastole (IVSd), which measures the thickness of the wall between the two ventricles during diastole. An EF% greater than 50% indicates heart functioning above half its capacity [17]. However, an EF% below 50% is considered abnormal, as it indicates that the heart is not functioning properly. A normal IVSd range is 0.6 cm to 1.2 cm, whereas an IVSd above 1.2 cm is considered abnormal [18].

### 2.4. Statistical Analysis

Demographic data were collected and analyzed with descriptive statistics using means and standard deviations for continuous variables and frequencies/proportions for dichotomous/polytomous variables. The H/CL ratios and PYP grades were collected and reported for all ten patients before and after treatment with inotersen or patisiran.

## 3. Results

### 3.1. Study Population

Ten patients were included in this study, with an average age of 63.80 ± 11.70 years (Table 1). The patients were predominantly males (*n* = 6, 60.0%). Most patients were of European descent (*n* = 6, 60.0%) and most patients were treated with patisiran (*n* = 7, 70.0%).

### 3.2. Comparisons of PYP Scan Results Before and After Treatment with TTR Silencer

Before treatment, all the patients were diagnosed as having ATTR-CM, since their PYP grade was a two or three and their H/CL ratio was ≥1.5 or more than 50%. A few patients had an H/CL ratio near 50% (*n* = 2, 20.0%), but they were diagnosed as having ATTR-CM (Table 2).

However, after treatment, half of the patients (*n* = 5, 50.0%) showed a reduction in their H/CL ratio in comparison to the H/CL ratio in their PYP scan that was taken before treatment. Furthermore, a few patients showed a reduction only in their PYP grade after treatment with a TTR silencer (*n* = 2, 20.0%). There were also few patients who showed a reduction post-treatment in both their H/CL ratio and PYP grade (*n* = 2, 20.0%). Lastly, there was one patient who did not show any improvements in either their H/CL ratio or PYP grade, as both values increased post-treatment.

### 3.3. Comparisons of IVSd and EF% Before and After Treatment with TTR Silencer

Before treatment, an interventricular septal end diastole (IVSd) of 1.2 cm or greater was suggestive of ATTR-CM. Furthermore, an ejection fraction percentage (EF%) greater than 50% was indicative of early-stage ATTR-CM; however, an EF% below 50% suggested late-stage ATTR-CM.

Before treatment, 8 out of 10 patients had an IVSd of 1.2 cm or greater, which is suggestive of ATTR-CM. Furthermore, 6 out of 10 patients had an EF% below 50%, which suggests late-stage ATTR-CM; however, the remaining patients had an EF% above 50%, which could suggest early-stage ATTR-CM (Table 3).

After treatment, only a few patients (*n* = 2, 20.0%) showed a reduction in their IVSd compared to their IVSd measurement before treatment. Half of the patients (*n* = 5, 50.0%) had the same IVSd level before and after treatment. However, a few patients (*n* = 3, 30.0%) showed a slight increase in their IVSd level after treatment. Additionally, half of the patients (*n* = 5, 50.0%) had an increase in their EF% compared to their EF% before treatment. However, a few patients (*n* = 4, 40.0%) showed a decrease in their EF% post-treatment, and one patient (*n* = 1, 10.0%) had the same EF% after treatment.

## 4. Discussion

To our knowledge, this is the first study to compare the cardiac effects in patients with ATTR before and after treatment with a TTR silencer. This study included ten patients with ATTR who had a mixed phenotype.

This study demonstrated different results. A total of 90.0% of the patients showed a decrease in their H/CL ratio and/or PYP grade. A reduction in at least one of these two parameters indicates improvements in terms of cardiac effects caused by amyloid deposits in the myocardium. This suggests that the TTR silencers used to treat these patients were able to silence the TTR mRNA and prevent it from translating into a TTR protein, which then led to a reduction in amyloid deposits in the myocardium. As a result, it was observed that the patients had a decrease in their signs of cardiomyopathy, as shown by the results of their PYP scan.

However, there was one patient who showed an increase in both their H/CL ratio and PYP grade after treatment with a TTR silencer. This could be due to the delay in obtaining a PYP scan post-treatment, as this patient underwent a PYP scan two years post-treatment. Since this disease is progressive and the treatments available would only help alleviate the signs and symptoms of cardiomyopathy for a certain period, it is possible that the TTR silencer was effective for a limited period of time; however, after this period, the TTR silencer may have been no longer effective in silencing the TTR mRNA and reducing the number of amyloid deposits in the myocardium [19]. Perhaps this patient could switch to the other TTR silencer available, as that could help alleviate their signs and symptoms of cardiomyopathy.

Furthermore, the patients’ echocardiograms showed varying results. A total of 50.0% of the patients showed an increase in their EF%, suggesting a reduction in amyloid accumulation in the myocardium post-treatment, as this improved the heart’s ability to pump blood out. However, 40.0% of the patients had a decrease in their EF%, which could suggest disease progression, despite being on treatment with a TTR silencer. There was also one patient who did not show any changes with their EF%, indicating disease stabilization, which is often preserved for patients with ATTR-CM.

The echocardiogram also assessed the IVSd for each patient, which showed different results. A total of 30.0% of the patients showed an increase in their IVSd, suggesting disease progression, as the amyloids continued to build up; however, 20.0% of the patients showed a decrease in their IVSd, indicating a regression of amyloid deposition and an improvement in the heart’s functioning. There were also 50.0% of the patients who did not show any changes in their IVSd, which tends to be the most common finding for patients with ATTR-CM, as this shows disease stabilization and that the wall thickness does not reverse dramatically.

Between the two types of TTR silencers, the Food and Drug Administration (FDA) reports that patisiran is effective at alleviating the symptoms of CM in patients with ATTR who have a mixed phenotype, as the clinical trial to determine the effectiveness of this treatment, APOLLO, showed a decrease in the morbidity and mortality regarding the cardiovascular phenotype [20]. Patisiran has not been approved by the FDA or Health Canada to treat patients who have only ATTR-CM, but patisiran could be explored to see whether it would be an effective treatment for patients with this phenotype as well. Since 70.0% of the patients were on patisiran, this could help explain why most patients showed an improvement in their PYP scan results and half of the patients showed an improvement in their EF% and a stable IVSd post-treatment.

On the other hand, inotersen has been approved by the FDA and Health Canada to treat patients with ATTR who have a mixed phenotype; however, its clinical trial, NEURO-TTR, showed that patients treated with this silencer tend to show improvements in their signs and symptoms of polyneuropathy [21]. The NEURO-TTR clinical trial also showed no difference in the signs of CM by analyzing echocardiographic parameters. This study showed otherwise; the patients showed improvements in CM, as 30.0% of the patients treated with inotersen showed a reduction in their H/CL ratio or PYP grade. A study on evaluating the effectiveness of inotersen at alleviating the symptoms of CM by analyzing the results of the PYP scan of patients with ATTR with a mixed phenotype could be explored.

Overall, although this study had a small sample size, it was able to determine that patients with ATTR who have a mixed phenotype and are treated with a TTR silencer tend to show an improvement in their CM. As a result, patients with ATTR-CM who are undergoing treatment should be monitored over time to see whether this treatment is effective for them.

### Study Limitations

This study may have been able to compare the cardiac effects before and after treatment with a TTR silencer, but there are some limitations. Although this study analyzed the cardiac effects of patients before and after treatment with inotersen or patisiran, not all cardiac parameters were analyzed, which includes measuring the levels of cardiac biomarkers, brain natriuretic peptide, and troponin I. This was due to limited data availability, as not all the patients had their levels of cardiac biomarkers measured before and/or after treatment with a TTR silencer. Furthermore, the sample size in this study was very small, as only 10 patients were included. The small sample size could be due to one of the following factors: (1) many patients who present with only ATTR-CM are treated with another treatment, tafamidis, as they would only be eligible for that treatment; (2) some patients refused follow-up; (3) some patients changed physicians; and (4) some patients passed away while the study was being conducted. In addition, the same treatment was not provided for all patients, as some received inotersen and others received patisiran. It is possible that one treatment is better at alleviating cardiac effects over the other, and this could explain why there was some variability in the results. Lastly, the timing of obtaining a PYP scan before treatment and another scan after treatment was not similar among all the patients. Some patients had a follow-up scan within the following year or two, whereas other patients had a follow-up scan about five years after their initial scan. Timing could have been a confounding factor when comparing scans before and after treatment.

## 5. Conclusions

Patients with ATTR who have a mixed phenotype could be treated with a TTR silencer, as it could help alleviate or stabilize their cardiac signs and symptoms, as shown in the results of their PYP scan and echocardiogram. A PYP scan and echocardiogram could be used to monitor patients over time while undergoing treatment to determine any improvements regarding their CM post-treatment. If the results indicate no improvements or a worsening of their symptoms, then physicians could switch to a more optimal treatment for the patients. Overall, it would be ideal for patients with ATTR with a mixed phenotype to undergo PYP scans and echocardiograms before and after treatment with a TTR silencer to observe any improvements in their CM post-treatment.

## Figures and Tables

**Table 1:** Demographics of patients.

Variable	*n* = 10
Age	63.80 ± 11.70
Sex:	
Male	6 (60.0%)
Female	4 (40.0%)
Ethnicity:	
Canadian	3 (30/0%)
European	6 (60.0%)
African	1 (10.0%)
Variant:	
Val142Ile	2 (20.0%)
Val30Met	1 (10.0%)
Val50Met	1 (10.0%)
Val150Leu	1 (10.0%)
Thr60Ala	1 (10.0%)
Thr80Ala	1 (10.0%)
Ile127Val	1 (10.0%)
Ile127Met	1 (10.0%)
Ile84Ser	1 (10.0%)
TTR Silencer:	
Inotersen	3 (30.0%)
Patisiran	7 (70.0%)

Values are expressed as means and standard deviations or frequencies and proportions. TTR = transthyretin.

**Table 2:** PYP ratios and PYP grades of the ten patients before and after treatment with a TTR silencer.

Patient	H/CL Ratio Before Treatment	PYP Grade Before Treatment	H/CL Ratio After Treatment	PYP Grade After Treatment
1	1.91	2	2.21	3
2	2.52	3	2.17	3
3	1.76	3	1.76	1
4	1.45	2	1.42	2
5	2.68	3	1.63	2
6	1.74	3	1.33	2
7	1.75	3	1.66	3
8	2.53	3	2.17	3
9	1.41	2	1.42	1
10	2.05	2	1.3	2

H/CL = heart-to-contralateral lung, PYP = pyrophosphate.

**Table 3:** IVSd and EF percentage of the ten patients before and after treatment with a TTR silencer.

Patient	IVSd Before Treatment	EF% Before Treatment	IVSd After Treatment	EF% After Treatment
1	1.5	34	1.6	37.5
2	2.1	47	2.1	34
3	2.3	57.5	1.4	52
4	1.0	55	1.1	57.5
5	1.5	66	1.4	71
6	1.3	57	1.3	59
7	1.5	40	1.8	44
8	1.4	47	1.4	44
9	1.6	63	1.6	62
10	1.0	60	1.0	60

IVSd = interventricular septal end diastole, EF% = ejection fraction percentage.

## Data Availability

All necessary data are included in the manuscript. All information obtained from the authors in this manuscript can be found in the literature.

## Abbreviations and Acronyms

ATTR	Transthyretin amyloidosis
TTR	Transthyretin
CM	Cardiomyopathy
PN	Polyneuropathy
PYP Scan	^99^Technetium pyrophosphate scintigraphy/^99^Tc-PYP scintigraphy
H/CL	Heart-to-contralateral lung ratio
ROI	Region of interest
mRNA	Messenger ribonucleic acid
PMCC	Peter Munk Cardiac Centre
FDA	Food and Drug Administration
